# CXCL12 Drives Reversible Fibroimmune Remodeling in Androgenetic Alopecia Revealed by Single-Cell RNA Sequencing

**DOI:** 10.3390/ijms26146568

**Published:** 2025-07-08

**Authors:** Seungchan An, Mei Zheng, In Guk Park, Leegu Song, Jino Kim, Minsoo Noh, Jong-Hyuk Sung

**Affiliations:** 1 Natural Products Research Institute, College of Pharmacy, Seoul National University, Seoul 08826, Republic of Korea; 2 Epi Biotech Co., Ltd., Incheon 21984, Republic of Korea; 3 New Hair Plastic Surgery Clinic, Seoul 06134, Republic of Korea

**Keywords:** androgenetic alopecia, androgen receptor, CXCL12, single-cell RNA sequencing, dermal fibrosis, stromal-immune interaction

## Abstract

Androgenetic alopecia (AGA) is a common form of hair loss characterized by androgen-driven tissue remodeling, including progressive follicular miniaturization and dermal fibrosis, which is accompanied by low-grade immune activation. However, the molecular mechanisms underlying this fibroimmune dysfunction remain poorly understood. Dermal fibroblasts (DFs) have been suggested as androgen-responsive stromal cells and a potential source of CXCL12, a chemokine implicated in fibroimmune pathology, but their precise role in AGA has not been fully established. In this study, we performed single-cell transcriptomic profiling of a testosterone-induced mouse model of AGA, with or without treatment of CXCL12-neutralizing antibody, to elucidate the pathological role of CXCL12 in mediating stromal-immune interactions. Our analysis suggested that DFs are the primary androgen-responsive population driving CXCL12 expression. Autocrine CXCL12-ACKR3 signaling in DFs activated TGF-β pathways and promoted fibrotic extracellular matrix deposition. In parallel, paracrine CXCL12-CXCR4 signaling reprogrammed Sox2^+^Twist1^+^ dermal papilla cells (DPCs) and promoted the accumulation of pro-fibrotic Trem2^+^ macrophages, contributing to impaired hair follicle regeneration. Notably, CXCL12 blockade attenuated these stromal and immune alterations, restored the regenerative capacity of DPCs, reduced pro-fibrotic macrophage infiltration, and promoted hair regrowth. Together, these findings identify CXCL12 as a central mediator of androgen-induced fibroimmune remodeling and highlight its potential as a therapeutic target in AGA.

## 1. Introduction

Androgenetic alopecia (AGA) is the most prevalent form of hair loss, characterized by the progressive miniaturization of hair follicles, particularly in the frontal and vertex scalp regions. This process leads to visible hair thinning and eventual baldness [1,2]. While often perceived as a cosmetic issue, AGA can have significant psychological consequences, including low self-esteem, anxiety, and depression [3]. Current treatment options, such as minoxidil and finasteride, are limited in efficacy and require long-term use to maintain hair density, highlighting the need for new, mechanism-based therapies [4].

The conventional understanding of AGA centers on androgen signaling, particularly through the action of dihydrotestosterone (DHT), a potent androgen that binds to the androgen receptor (AR) in dermal papilla cells [5,6,7,8,9]. AR activation alters the expression of key regulatory genes, leading to shortened anagen duration, impaired hair cycling, and follicular regression [10]. However, growing evidence suggests that the AGA pathogenesis involves more than hormonal effects alone. Histological studies of balding scalp have revealed notable dermal fibrosis, extracellular matrix (ECM) accumulation, and immune cell infiltration [11,12,13], suggesting broader dysregulation of the skin microenvironment involving stromal and immune compartments.

One molecule that may bridge androgen signaling with fibrotic and immune remodeling is CXCL12, also known as stromal cell-derived factor 1 (SDF-1). CXCL12 is a chemokine abundantly expressed in dermal fibroblasts and signals primarily through CXCR4 and ACKR3 (also known as CXCR7) [14,15,16,17]. These receptors mediate a wide range of biological processes, including tissue fibrosis, immune cell recruitment, and stem cell homing [18,19]. Previous work from our group has shown that testosterone and DHT increase CXCL12 expression in the scalp and that blocking CXCL12 promotes hair regrowth by reducing AR expression in DPCs [7]. Nevertheless, the specific cellular sources of CXCL12, the downstream target cell types, and the overall architecture of CXCL12-driven signaling in AGA remain poorly defined [17].

To address this issue, we performed single-cell RNA sequencing (scRNA-seq) in a testosterone-induced mouse model of AGA, with and without treatment using a CXCL12-neutralizing antibody. This approach enabled us to resolve the cellular heterogeneity of the skin, identify androgen-responsive cell populations, and delineate the transcriptional programs underlying AGA progression and its reversal. Our findings demonstrate that dermal fibroblasts are the primary androgen-responsive cells that produce CXCL12, which acts both autocrinally to promote fibrosis via TGF-β signaling and paracrinally to reprogram neighboring DPCs. In parallel, CXCL12-CXCR4 signaling supports the expansion of pro-fibrotic macrophages.

## 2. Results

### 2.1. Single-Cell Transcriptome Reveals Altered Skin Cell Composition in the AGA Model

**Figure 1:**
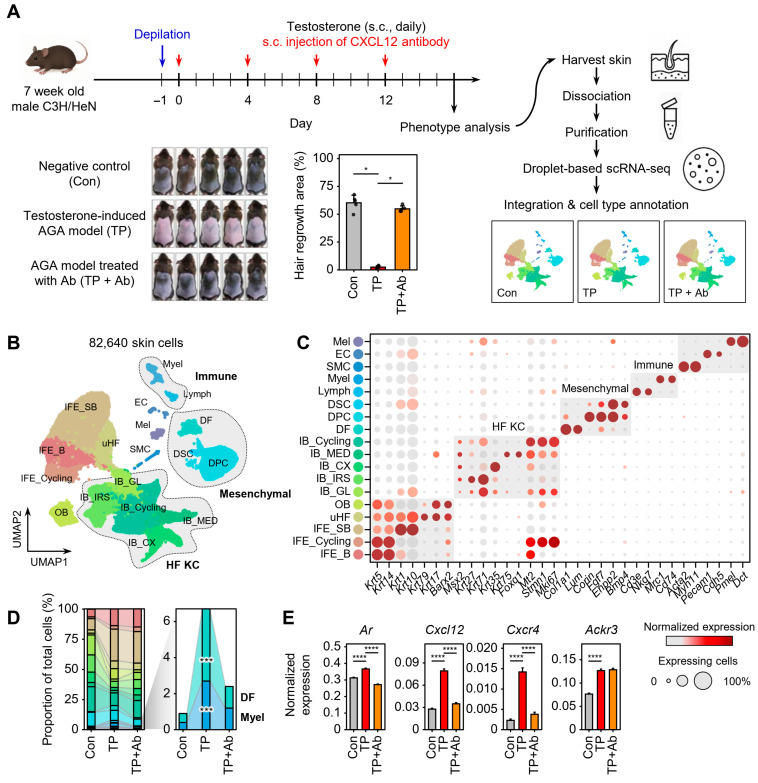
Single-cell transcriptomic profiling of androgenetic alopecia. (**A**) Experimental overview. AGA was induced with testosterone propionate (TP), treated with a CXCL12-neutralizing antibody (TP + Ab), and analyzed by droplet-based scRNA-seq. Hair regrowth area was quantified using the Trainable Weka Segmentation plugin in Fiji. Statistical significance assessed using Wilcoxon test; * *p* < 0.05. (**B**) UMAP plot showing clustering of all skin cells from Con, TP, and TP + Ab groups, annotated by cell type. (**C**) Dot plot of representative marker genes used to determine cell types. IFE, interfollicular epidermis keratinocyte (IFE_B, basal IFE; IFE_SB, suprabasal IFE); uHF, upper hair follicle keratinocyte; OB, outer bulge; IB, inner bulge (GL, germinative layer; IRS, inner root sheath; CX, cortex; MED, medulla); DF, dermal fibroblast; DPC, dermal papilla cell; DSC, dermal sheath cup cell; Lymph, lymphocyte; Myel, myeloid cell; SMC, smooth muscle cell; EC, endothelial cell; Mel, melanocyte. (**D**) Bar plot of cell type proportions across groups. Significance was assessed using the binomial test; *** *p* < 0.001. (**E**) Mean (±standard error) expression levels of AR, CXCL12, CXCR4, and ACKR3 across groups. Statistical significance assessed using Wilcoxon test; **** *p* < 0.0001.

We previously demonstrated that CXCL12 plays a critical role in the pathogenesis of AGA in a testosterone-induced mouse model and that a neutralizing antibody against CXCL12 (CXCL12 Ab) rescues hair loss in this context [14]. To uncover the cellular and molecular mechanisms underlying AGA and the therapeutic impact of CXCL12 blockade, we performed scRNA-seq on dorsal skin from three groups: control mice, testosterone propionate (TP)-treated AGA model mice, and TP-treated mice administered a CXCL12-neutralizing antibody (TP + Ab) [20]. TP treatment induced pronounced hair loss, which was significantly attenuated by CXCL12 Ab co-treatment (Figure 1A). From 82,640 high-quality single-cell transcriptomes, we identified 18 distinct skin cell types (Figure 1B and Figure S1), including keratinocytes, dermal fibroblasts (DFs), dermal papilla cells (DPCs), melanocytes, and immune cells such as lymphocytes and myeloid cells (Figure 1C). While the overall cellular landscape was preserved, TP-treated skin showed marked expansion of DFs and myeloid cells (8.0- and 6.8-fold, respectively; *p* < 0.001), which was substantially restored by CXCL12 Ab treatment (Figure 1D).

At the transcript level, TP treatment led to significantly increased gene expression of AR (androgen receptor), CXCL12 (the therapeutic target), and its canonical receptor CXCR4 by 1.2-, 2.8-, and 6.2-fold, respectively, compared with control (Figure 1E). Importantly, CXCL12 Ab treatment markedly downregulated the expression of AR, CXCL12, and CXCR4, supporting the efficacy of Ab treatment in suppressing CXCL12-driven signaling. In contrast, expression of ACKR3, which encodes the alternative receptor CXCR7, was elevated in TP-treated skin (1.7-fold; *p* < 0.001) but remained unaffected by Ab treatment, suggesting possible receptor-specific regulation or involvement in distinct signaling pathways (Figure 1E).

### 2.2. Androgen Induces Transcriptional Reprogramming Reversed by CXCL12 Blockade

**Figure 2:**
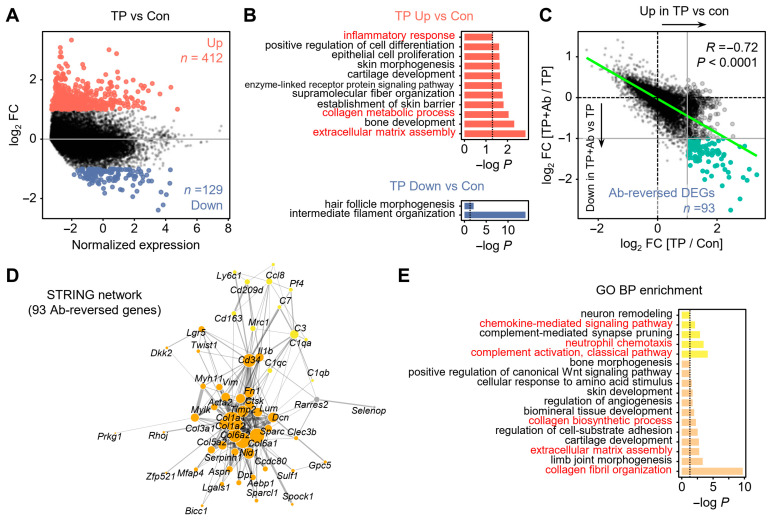
Global transcriptomic change by CXCL12 blockade in AGA model. (**A**) MA plot of TP vs. Con with DEGs defined as |log_2_ fold change| ≥ 1. (**B**) GO biological process enrichment of DEGs upregulated or downregulated by TP treatment (adjusted *p* < 0.05). (**C**) Correlation between log_2_ FC values from TP vs. Con and TP + Ab vs. TP comparisons, with a green line indicating the linear regression trend. Pearson’s R and *p* value are shown. (**D**) STRING network of Ab-reversed genes (DEGs upregulated in TP vs. Con and downregulated in TP + Ab vs. TP). (**E**) GO BP enrichment of Ab-reversed DEGs grouped by communities detected in STRING network (adjusted *p* < 0.05).

To assess global transcriptional changes, we next performed pseudobulk differential expression analysis by aggregating single-cell profiles per experimental group (Figure 2). Comparison between TP-treated and control skin revealed 412 significantly upregulated DEGs (log_2_ fold change ≥ 1) and 129 downregulated genes (Figure 2A). In contrast, the TP + Ab group, compared with TP, showed only eight upregulated DEGs (Figure S2A). Gene ontology (GO) enrichment analysis of TP-upregulated DEGs revealed significant overrepresentation of biological processes related to inflammation, collagen metabolism, and ECM assembly, which are hallmark features of AGA molecular pathology (Figure 2B). Conversely, genes downregulated by TP were enriched for hair follicle morphogenesis, supporting the TP-treated AGA model’s reliability in capturing transcriptional signatures of hair loss (Figure 2B). Notably, Ab treatment restored the expression of hair follicle morphogenesis-related genes (Figure S2B) while suppressing TP-induced activation of ECM assembly, collagen fibril organization, and chemokine signaling pathways, highlighting the therapeutic effects of CXCL12 blockade (Figure S2C).

To quantitatively assess the reversal effect of CXCL12 inhibition, we evaluated the correlation between gene expression changes in TP vs. control and those in TP + Ab vs. TP. A strong negative correlation (*R* = −0.72, *p* < 0.0001) indicated that many androgen-dysregulated genes were reversed by Ab treatment (Figure 2C). To systematically investigate this reversal effect, we identified a set of ‘Ab-reversed DEGs’, defined as genes significantly upregulated in TP compared with control but downregulated in TP + Ab compared with TP. Among these, we identified 93 Ab-reversed DEGs [21], which represent a core gene set associated with the effect of CXCL12 inhibition (Figure 2D). These genes were significantly enriched for pathways involving chemokine-mediated signaling, complement activation, collagen biosynthesis, ECM organization, and collagen fibril assembly (Figure 2E). Gene set enrichment analysis (GSEA) based on ranked fold change differences further confirmed the involvement of inflammatory responses, collagen and ECM remodeling, and myeloid cell activation and migration in the AGA pathogenesis, all of which were diminished by CXCL12 Ab treatment (Figure S3).

### 2.3. DFs Are Key Androgen-Responsive Cells Driving CXCL12 Expression

**Figure 3:**
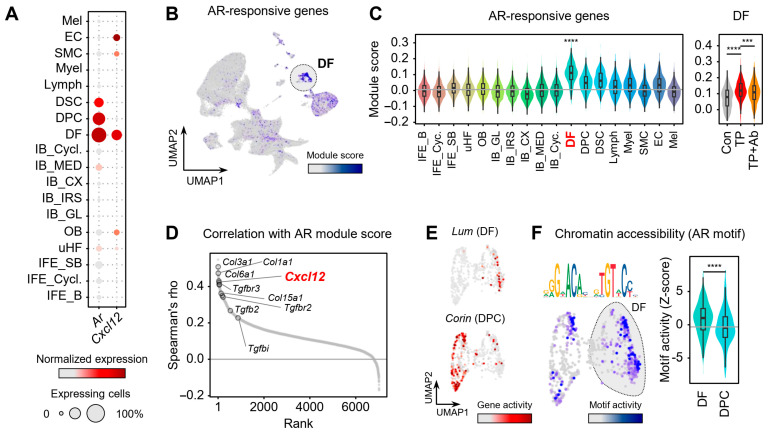
Dermal fibroblasts as the primary androgen-responsive CXCL12 source. (**A**) Dot plot of AR and CXCL12 expression across cell types. (**B**) UMAP colored by AR module score, calculated with curated AR target genes from TFLink database. (**C**) Violin plots showing AR module score across groups and cell types. Wilcoxon test; *** *p* < 0.001, **** *p* < 0.0001. (**D**) Correlation between AR module score and expression of all other genes within the DF population, ranked by Spearman’s rho. (**E**) UMAP of mouse skin scATAC-seq (GSE227784) with DF and DPC annotated by marker gene activity. (**F**) AR motif accessibility was quantified by chromVAR. Sequence logo represents the AR motif (MA0007.3) used for accessibility analysis. Wilcoxon test; **** *p* < 0.0001.

To identify the cell types responsible for CXCL12 upregulation in AGA, we first examined the expression of the androgen receptor gene AR across skin cell types (Figure 3A). Notably, AR expression was markedly enriched in mesenchymal lineages, particularly DFs. To assess AR signaling activity, we next calculated AR module scores using a curated set of AR-responsive genes. DFs showed the highest scores among all cell types (*p* < 0.001), which were elevated by TP treatment and were significantly reduced upon CXCL12 Ab administration (Figure 3B,C). These findings suggest that DFs are the principal androgen-responsive population in AGA and that CXCL12 blockade attenuates androgen-driven transcriptional activity in these cells. Cell–cell interaction analysis further supported that mesenchymal lineages were the predominant recipients of testosterone-mediated signaling (Figure S4A) [22], while DFs in TP-treated mice showed strong activation of these pathways, which was markedly diminished by CXCL12 Ab treatment (Figure S4B).

We next sought to identify downstream transcriptional targets of AR by correlating module scores with gene expression levels in DFs (Figure 3D). Notably, Cxcl12 emerged as one of the top genes positively correlated with AR activity (*R* = 0.35, *p* < 0.0001), suggesting a functional link between androgen signaling and CXCL12 transcription in DFs (Figure S5). Although DPCs and dermal sheath cup cells also express AR, they showed weaker AR module activity. We hypothesized that differential chromatin accessibility might explain their reduced responsiveness. To test this, we analyzed public single-cell ATAC-seq data from mouse skin (GSE227784) and identified DF and DPC populations based on gene activity profiles of known markers (Figure 3E and Figure S6) [23]. Interestingly, using chromVAR analysis [24], we found AR motifs to be significantly more accessible in DFs than in DPCs, reflected by a higher deviation Z-score (DF median = 1.0 vs. DPC = −0.44) (Figure 3F). Together, DFs emerge as central effectors of androgen-driven CXCL12 expression, with epigenetic regulation that contributes to their responsiveness, attenuated by CXCL12 blockade.

### 2.4. CXCL12 Acts in an Autocrine Manner in DFs to Activate TGF-β Signaling and ECM Remodeling

**Figure 4:**
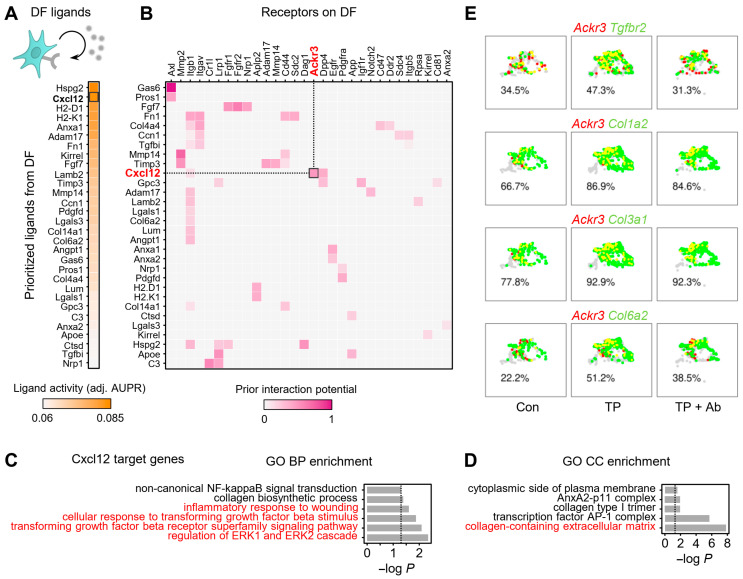
Autocrine CXCL12-ACKR3 signaling in fibroblasts. (**A**,**B**) NicheNet analysis identifying CXCL12 among top ligands based on differential expression between TP and control: (**A**) ligand activity (adjusted AUPR) and (**B**) ligand-receptor prior interaction potential. (**C**,**D**) GO enrichment analysis of predicted CXCL12 targets for biological processes (**C**) and cellular components (**D**). (**E**) Co-expression of ACKR3 and TGF-β/ECM genes in DF subset; red = Ackr3^+^, green = specified gene-positive, and yellow = double-positive.

To explore how CXCL12 upregulation in DFs contributes to the AGA microenvironment, we next performed cell–cell interaction analysis (Figure 4). Among the differentially expressed ligands in DFs (defined as >30% expression and log_2_ fold change >0.5), CXCL12 ranked as the second most potent ligand based on predicted ligand activity, highlighting its central role in intercellular communication within AGA skin (Figure 4A). We then identified ACKR3 as a key receptor for CXCL12-driven autocrine signaling in DFs, supported by ligand-receptor interaction analysis using NicheNet [25], which showed the highest communication probability for the DF-autocrine CXCL signaling network (Figure 4B and Figure S7). In addition, the proportion of Ackr3^+^ DFs increased in the TP group, then decreased following CXCL12 Ab treatment. Downstream of the CXCL12-ACKR3 signaling axis, predicted target genes included *Col16a1*, *Mmp2*, *Anxa1*, and *Tgfbr2* (Figure S8A). GO enrichment analysis of these targets revealed significant associations with fibrosis-related biological processes, including TGF-β response and ECM assembly (Figure 4C). Notably, in the GO cellular component category, these genes were enriched in terms related to collagen-containing ECM, supporting a pro-fibrotic role for CXCL12-mediated signaling in AGA (Figure 4D).

We further observed that several TGF-β signaling components (e.g., *Tgfbr2*) and ECM genes (*Col1a2*, *Col3a1*, and *Col6a2*) exhibited strong co-expression with ACKR3 in DFs (Figure 4E). This co-expression was elevated in the TP-treated AGA group compared with controls and reduced after CXCL12 Ab treatment. Expression levels of these genes followed a similar pattern, with significant upregulation in the TP group and reversal upon Ab treatment, indicating that CXCL12-ACKR3 signaling promotes TGF-β-driven collagen production in an autocrine manner (Figure S8B). These results suggest that androgen-induced CXCL12 expression in DFs activates an autocrine loop through ACKR3, driving TGF-β signaling and ECM deposition, which contributes to fibrotic remodeling in AGA skin.

### 2.5. CXCL12-CXCR4 Signaling Alters DPC Subpopulations and Promotes ECM Remodeling in AGA

**Figure 5:**
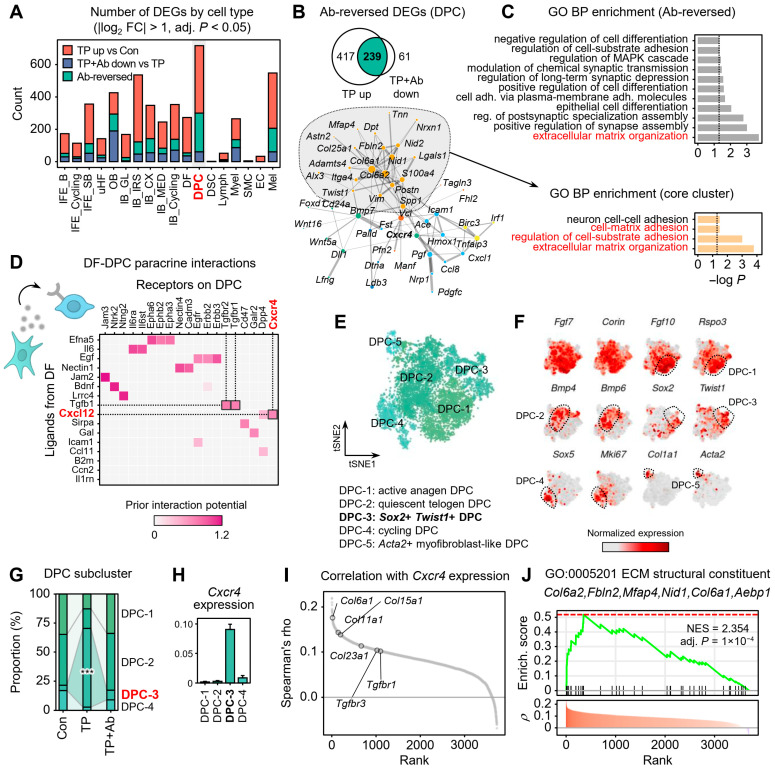
Paracrine CXCL12-CXCR4 signaling in dermal papilla cells. (**A**) Number of DEGs from TP vs. Con and TP + Ab vs. TP comparisons, across all cell types. (**B**) STRING network of 239 Ab-reversed DEGs in DPCs. (**C**) GO enrichment for all Ab-reversed DEGs (top) and largest STRING community (orange, bottom). (**D**) NicheNet ligand-receptor prior interaction potential between DFs and DPCs based on differential gene expression between TP and control. (**E**) tSNE plot showing five DPC subclusters: DPC-1 (active anagen DPC), DPC-2 (quiescent telogen DPC), DPC-3 (Sox2^+^Twist1^+^ DPC), DPC-4 (cycling DPC), and DPC-5 (Acta2^+^ myofibroblast-like DPC). (**F**) tSNE plots colored by expression of marker genes for each subcluster. (**G**) Subcluster proportions across groups; binomial test *** *p* < 0.001. (**H**) Mean (±standard error) expression levels of CXCR4 across DPC subclusters. (**I**) Spearman correlation between CXCR4 expression and expression levels of all other genes in DPCs. (**J**) GSEA of genes positively correlated with CXCR4 expression.

While CXCL12 signaling promotes fibrosis via autocrine effects in DFs, we next examined its potential paracrine effects on DPCs, which play a central role in hair cycling regulation. To identify cell populations most affected by androgen and rescued by CXCL12 blockade, we analyzed Ab-reversed DEGs in each cell type (Figure 5A). DPCs exhibited the largest number of Ab-reversed DEGs (n = 239), suggesting that they are a key target of both androgen-induced pathogenesis and CXCL12-mediated reversal (Figure 5B). Functional enrichment of these Ab-reversed DEGs in DPCs revealed strong associations with ECM organization, cell-substrate adhesion, and cell-matrix adhesion, patterns also observed in DFs (Figure 5C). Additionally, several cell-type-specific Ab-reversed DEGs, including those in keratinocytes, also showed significant changes in ECM assembly, collagen fibril organization, and ECM organization in the AGA niche (Figure S9). Notably, CXCL12-CXCR4 signaling from DFs to DPCs was enriched in TP compared with control (Figure 5D). In addition, CXCR4 expression was significantly elevated in DPCs of TP-treated mice (12.7-fold compared with control) and returned to near-baseline levels following CXCL12 Ab treatment, with limited changes in ACKR3 (Figure S10A).

To dissect DPC heterogeneity, we performed unsupervised subclustering of DPCs (Figure 5E), revealing five distinct subpopulations: (1) Fgf10^+^ Rspo3^+^ active anagen DPCs, (2) Bmp4^+^ Bmp6^+^ quiescent telogen DPCs, (3) Sox2^+^Twist1^+^ DPCs, (4) Mki67^+^ proliferating DPCs, and (5) Acta2^+^ myofibroblast-like DPCs (Figure 5F). While all clusters expressed canonical DPC markers such as *Fgf7* and *Corin*, each exhibited distinct functional characteristics. The active anagen cluster (DPC-1) strongly expressed *Fgf10* and *Rspo3*, known to promote epithelial proliferation via Wnt signaling [26,27]. The quiescent telogen DPC cluster (DPC-2) was marked by *Bmp4* and *Bmp6*, which inhibit telogen-to-anagen transition [28,29]. The cluster DPC-3 exhibited low expression of *Fgf10* and *Rspo3* but was marked by high levels of *Sox2* and *Twist1*, suggesting a distinct identity from classical anagen or quiescent DPCs.

Notably, the Sox2^+^Twist1^+^ subpopulation (DPC-3) was significantly enriched in TP-treated mice (*p* < 0.001; Figure 5G) and expressed the highest CXCR4 levels (Figure 5H). Genes correlated with CXCR4 included *Col6a1*, *Col15a1*, and other ECM components in DPCs (Figure 5I), and enrichment analysis also suggested their involvement in the GO term “ECM structural constituent” (Figure 5J). These Sox2^+^Twist1^+^ DPCs also exhibited elevated TGF-β receptor (*Tgbfr1*, *Tgbfr2*, and *Tgbfr3*) and collagen (*Col6a1* and *Col6a2*) expression, suggesting a pro-fibrotic role (Figure S10B,C). CellChat analysis further supported this interpretation, showing the stronger DF-to-DPC collagen signaling in AGA compared with control (Figure S11). In sum, androgen-activated DFs secrete CXCL12, signaling to Sox2^+^Twist1^+^ DPCs via CXCR4 to drive ECM remodeling and suppress hair induction, with CXCL12 neutralization attenuating these effects.

### 2.6. CXCL12-CXCR4 Signaling Promotes Retention of Trem2^+^ Macrophages and Contributes to Fibrosis in AGA

scRNA-seq analysis revealed that TP treatment expanded myeloid populations and upregulated genes linked to their activation and migration, which were reversed by CXCL12 Ab treatment (Figure 1, Figure 2 and Figure S12). In addition, inflammatory response-related genes were significantly upregulated in myeloid cells of the TP group, suggesting that those cells are major contributors to the inflammatory milieu in AGA (Figure S13).

**Figure 6:**
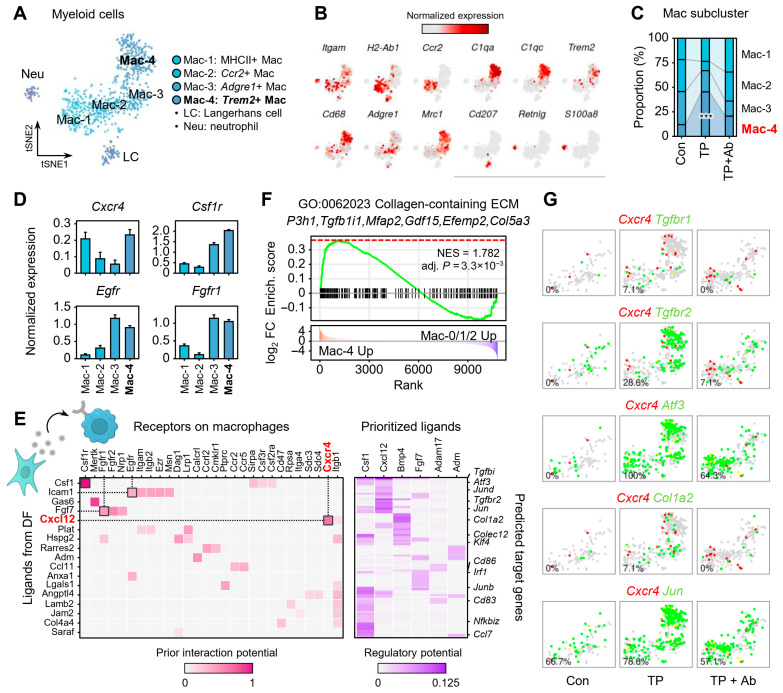
CXCL12-driven retention of Trem2^+^ macrophages. (**A**) tSNE plot of myeloid subset, clustered into subclusters: Mac-1 (MHCII^+^ antigen-presenting Mac), Mac-2 (Ccr2^+^ recruited monocyte-like Mac), Mac-3 (Adgre1^+^ resident Mac), Mac-4 (Trem2^+^ Mac), LC (Cd207^+^ Langerhans cell), and Neu (Retnlg^+^ S100a8^+^ neutrophil). (**B**) tSNE plots colored by expression of marker genes for each subcluster. (**C**) Subcluster proportions across groups; binomial test *** *p* < 0.001. (**D**) Mean (±standard error) expression levels of *Cxcr4*, *Csf1r*, *Egfr*, and *Fgfr1* across macrophage subclusters. (**E**) NicheNet-predicted ligand-receptor prior interaction potential and regulatory potential for target genes between DFs and macrophages, based on TP vs. Con comparison. (**F**) GSEA of DEGs enriched in Trem2^+^ macrophages (Mac-4). (**G**) Co-expression of *Cxcr4* and fibrosis-related genes (Tgfbr1/2, *Atf3*, *Col1a2*, and *Jun*) in macrophages; red = Cxcr4^+^, green = specified gene-positive, and yellow = double-positive.

To investigate this further, we next focused on the myeloid compartment in our single-cell dataset (Figure 6). Subclustering of myeloid cells identified distinct macrophage subsets: (1) MHCII^+^ antigen-presenting macrophages, (2) Ccr2^+^ monocyte-like macrophages, (3) Adgre1^+^ (F4/80) tissue-resident macrophages, and (4) Trem2^+^ macrophages (Figure 6A,B). Specifically, Mac-1 and Mac-2 expressed *Ccr2*, a marker of recruited monocyte-like macrophages, with Mac-1 highly expressing *H2-Ab1*, which encodes the MHC class II molecule. In contrast, Mac-3 and Mac-4 expressed *Adgre1*, a marker of resident macrophages [30]. Notably, Mac-4 showed high expression of complement-related genes (*C1qa*, *C1qc*), *Cd68*, and the immune-regulatory marker Trem2, distinguishing it from Mac-3.

Notably, a significant expansion of the Trem2^+^ subset (Mac-4) following TP treatment was observed, which was reduced by Ab administration (*p* < 0.001; Figure 6C). These Trem2^+^ macrophages showed high CXCR4 expression (Figure 6D and Figure S12C). In addition, Trem2^+^ Mac-4 showed an M2-like anti-inflammatory state, while MHCII^+^ Mac-1 displayed a more pro-inflammatory, M1-like profile (Figure S14). Importantly, Cxcr4 was expressed in both M1-like Mac-1 and M2-like Mac-4 subsets (Figure 6D), but only the Trem2^+^ Mac-4 population expanded in AGA, suggesting that additional DF-derived signals cooperate with CXCL12 to selectively promote the retention of this subset.

To examine this possibility, we performed NicheNet ligand-receptor analysis of interactions between DFs and macrophages [25]. In addition to CXCL12-CXCR4, other predicted interaction pairs included CSF1-CSF1R, FGF7-FGFR1, and ICAM1-EGFR (Figure 6E). Notably, transcription of Csf1r, Fgfr1, and Itgam was more selectively enriched in Trem2^+^ cells, suggesting they may contribute to the persistence of this subset in AGA (Figure 6D). Target gene analysis revealed limited overlap between downstream signaling pathways: CXCL12 likely regulates fibrosis-related genes such as *Tgfbi*, *Atf3*, *Tgfbr2*, *Col1a2*, and *Jun*, whereas CSF1 and FGF7 were associated with distinct targets like *Junb*, *Nfkbiz*, or *Irf1*. This suggests that multiple DF-derived signals complementarily shape the Trem2^+^ macrophage niche (Figure 6E). GSEA of Trem2^+^ macrophages showed enrichment of ECM organization (Figure 6F), indicating that androgen-induced CXCL12 signaling supports the retention and activation of Trem2^+^ macrophages via CXCR4, contributing to ECM remodeling and fibrosis in coordination with DFs and DPCs. Consistently, *Cxcr4* co-expression with genes such as *Tgfbr1*, *Tgfbr2*, *Atf3*, *Col1a2*, and *Jun* further implicates this pathway in fibrotic remodeling (Figure 6G). Taken together, CXCL12 Ab prevents pro-fibrotic Trem2^+^ macrophage accumulation, aiding the restoration of a hair growth-supportive skin microenvironment.

## 3. Discussion

AGA has long been understood primarily as a hormone-mediated condition driven by DHT acting through AR in DPCs [31,32]. However, this paradigm does not fully explain the progressive fibrosis, immune cell infiltration, and microenvironmental changes observed in balding scalp tissue [5,11,12]. Our study challenges this traditional view by identifying CXCL12 as a key intermediary linking androgen signaling to broader stromal and immune remodeling events in AGA.

Using scRNA-seq in a testosterone-induced mouse model of AGA, we found that DFs, rather than DPCs, are the most androgen-responsive cell population. These fibroblasts exhibit elevated AR module activity and selectively accessible AR motifs at the chromatin level. Importantly, they upregulate CXCL12, a chemokine that mediates both autocrine and paracrine signaling. Autocrine CXCL12-ACKR3 signaling within DFs activates TGF-β pathways and promotes ECM deposition, contributing to dermal fibrosis [33,34]. Simultaneously, CXCL12 acts paracrinically on CXCR4-expressing DPCs, specifically a Sox2^+^Twist1^+^ subpopulation, reprogramming them toward a fibrotic, low-inductive state that diminishes their capacity to maintain follicular stem cells and initiate anagen.

Beyond the stromal and follicular compartments, our study also reveals a pivotal role for CXCL12 in modulating the immune microenvironment. We observed a marked expansion of Trem2^+^ macrophages in AGA skin, which express high levels of CXCR4 and genes associated with ECM remodeling and immunosuppression [35,36]. These macrophages resemble alternatively activated (M2-like) subsets seen in other fibrotic conditions and may contribute to the persistence of the fibrotic niche. Cell–cell communication analyses suggest that DF-derived CXCL12, in combination with CSF1 and FGF7, supports the retention and activation of these macrophages.

Therapeutically, the administration of a CXCL12-neutralizing antibody effectively reversed transcriptomic changes in DFs, DPCs, and macrophages; restored gene signatures associated with regenerative capacity; and promoted visible hair regrowth in the mouse model. This provides compelling evidence for targeting CXCL12 as a promising treatment strategy for AGA. In contrast to current FDA-approved options like minoxidil and finasteride, which act on vascular tone and androgen metabolism, respectively, CXCL12 inhibition operates at the intersection of stromal, follicular, and immune pathways, offering a mechanism-based approach to restoring dermal homeostasis [4].

Furthermore, our findings position CXCL12 as a critical node that integrates hormonal, fibrotic, and immune signaling in the skin. The spatial convergence of AR signaling, TGF-β activation, and myeloid cell recruitment around fibroblast-derived CXCL12 suggests a self-reinforcing pathological loop. Interrupting this loop not only halts disease progression but also restores the cellular balance necessary for hair regeneration.

These insights also have broader implications beyond AGA. Similar CXCL12-mediated fibroimmune mechanisms may be operative in other chronic inflammatory or fibrotic skin diseases such as lichen planopilaris, chronic graft-versus-host disease, or even scleroderma [37,38,39]. Thus, CXCL12 inhibition could represent a shared therapeutic strategy across multiple dermatoses characterized by aberrant stromal-immune interactions.

While our study identifies CXCL12 as a central mediator of fibroimmune remodeling in AGA and establishes proof-of-concept for therapeutic intervention, several limitations warrant consideration. The testosterone-induced mouse model effectively recapitulates key pathological features of AGA but may not fully capture species-specific differences in skin architecture and immune regulation. Functional validation of the identified CXCL12-responsive cell populations, including Sox2^+^Twist1^+^ DPCs and Trem2^+^ macrophages, will require future lineage-tracing and targeted perturbation studies. Additionally, the long-term safety profile of CXCL12 blockade remains to be fully evaluated, particularly given the chemokine’s roles in stem cell trafficking and immune homeostasis. Finally, while we focused on the CXCL12 axis, other androgen-regulated stromal or immune mediators may also contribute to AGA pathogenesis and represent promising avenues for future investigation. Together, these considerations underscore the need for further mechanistic and translational studies to fully realize the therapeutic potential of targeting fibroimmune circuits in chronic skin diseases.

## 4. Materials and Methods

### 4.1. Ethics Statement

The animal study was approved by the Institutional Animal Care and Use Committee of Yonsei University (IACUC-202302-1636-01) and was conducted in accordance with local legislation and institutional guidelines. No adverse events were observed during the study. Procedures were not expected to cause pain or distress; all injections were performed under standard handling protocols to minimize stress. Humane endpoints were not predefined, as no morbidity or mortality was anticipated based on prior experience with this protocol. Mice were housed in individually ventilated cages, with a 12:12 light-dark cycle, ad libitum access to food and water, and nesting material provided for environmental enrichment.

### 4.2. AGA Animal Model

To induce AGA, the dorsal skin of 7-week-old male C3H mice (n = 45) was shaved, followed by subcutaneous injection of testosterone propionate (TP; 0.5 mg/day). A CXCL12-neutralizing antibody (5 μg) was administered via subcutaneous injection twice weekly for two consecutive weeks. Mice were assigned to three groups (n = 15 per group): control (no TP; vehicle), TP-only (AGA mouse model), and TP + CXCL12-neutralizing antibody. Animals were manually randomized to ensure balanced group sizes and minimize confounding by treatment order. The experimental unit was defined as each individual mouse. No animals or samples were excluded from the study. All mice that received the planned treatments were included in the final analysis. Hair regrowth was quantitatively evaluated using the Trainable Weka Segmentation plugin in Fiji ImageJ version 1.54p (https://imagej.net/software/fiji/ (accessed on 6 July 2025)), based on pixel classification of shaved versus regrown areas [40].

### 4.3. Preparation of Single-Cell Suspension

Skin tissues were collected and pooled from the dorsal region of mice (n = 15 per group) and processed as previously described [20]. Samples were pooled, minced, and enzymatically digested in 0.7 mg/mL collagenase D (Sigma-Aldrich). The resulting suspension was filtered through cell strainers to remove aggregates. Red blood cells were lysed using ACK Lysing Buffer (Gibco, Waltham, MA, USA), and debris was removed via density gradient centrifugation with Percoll (Sigma-Aldrich, St. Louis, MS, USA). The single-cell suspension was immediately used for scRNA-seq.

### 4.4. Droplet-Based scRNA-Seq

Single-cell suspensions were processed using the Chromium GEM-X Single Cell 3′ RNA v4 kit (10× Genomics, Pleasanton, CA, USA), following the manufacturer’s protocol. Briefly, cells were partitioned into Gel Beads-in-Emulsion (GEMs) containing barcoded oligonucleotides. Poly (dT) primers captured polyadenylated mRNA from individual cells, enabling the synthesis of barcoded cDNA. The resulting cDNA was amplified to construct 3′ gene expression libraries and sequenced on an Illumina platform at Macrogen (Seoul, Republic of Korea). Raw BCL files were demultiplexed into FASTQ format using Cell Ranger v8.0.1 (10× Genomics), and reads were aligned to the mouse reference genome (mm10-2020-A) using the cellranger count pipeline, which also performed UMI quantification and cell barcode filtering. Investigators performing library construction and RNA-seq were blinded to group assignments.

### 4.5. Single-Cell Transcriptome Analysis

We analyzed 107,662 skin cells from three experimental groups. Preprocessing, clustering, and visualization were performed using the Seurat R package (v5.0.3) as previously described [20,41,42]. Cells expressing fewer than 200 genes or more than 8000 genes or with mitochondrial gene content exceeding 10% were excluded. Doublets were identified and removed using DoubletFinder (v2.0.4) with default parameters [43]. The count matrix was normalized, and the top 2000 highly variable genes were selected for scaling; then, the matrices were integrated using the IntegrateData module of Seurat. Clustering was performed using a shared nearest-neighbor approach followed by the Louvain algorithm. Dimensionality reduction was performed using UMAP (Uniform Manifold Approximation and Projection) or tSNE (t-distributed Stochastic Neighbor Embedding). Clusters were annotated manually based on canonical and literature-defined marker genes [20,44].

### 4.6. Differential Expression and Functional Enrichment

Pseudobulk differential expression analysis was performed using the PseudobulkExpression module in Seurat by aggregating gene counts per sample. Genes with an absolute log_2_ fold change ≥1 between groups were defined as differentially expressed genes (DEGs), which were further analyzed using R package STRINGdb (v2.16.4) to identify putative protein–protein interaction networks [21]. Community detection was carried out using the igraph R package (v2.0.3). Functional enrichment was performed using R packages gprofiler2 (v0.2.3) and fgsea (v1.30.0). Cell-type-specific DEGs were identified using the FindMarkers function in Seurat [41].

### 4.7. Cell–Cell Communication Analysis

Intercellular signaling was inferred using the CellChat R package (v2.1.1), which predicts ligand-receptor interactions between cell types based on known signaling pathways and evaluates the strength of communication networks [22]. This approach enabled us to identify major signaling sources and targets within the AGA skin microenvironment and to visualize how cell–cell communication was altered by treatment. To complement this, NicheNet (v2.2.0) was employed to prioritize ligand-target gene relationships [25]. Unlike CellChat, which focuses on receptor engagement, NicheNet integrates prior knowledge of signaling and gene regulatory networks with observed transcriptional responses to infer which ligands from sender cells most likely regulate gene expression changes in receiver cells.

### 4.8. Module Score

Module scores for AR-responsive genes, inflammatory response signatures, and macrophage M1/M2 polarization were calculated using the AddModuleScore function in Seurat [41]. AR target genes were obtained from the TFLink database, and the Hallmark Inflammatory Response gene set was retrieved from MSigDB. These module scores were used to quantify transcriptional activity in each cell.

### 4.9. Single-Cell Assay for Transposase-Accessible Chromatin Using Sequencing (scATAC-Seq) Analysis

To investigate lineage-specific chromatin accessibility in mesenchymal cell types, we analyzed publicly available mouse skin scATAC-seq data (GSE227784) from 12-week-old mice [23]. Raw reads were processed using Cell Ranger ATAC v2.1.0, and peak-by-barcode matrix was loaded and processed using the Signac (v1.14.0) and Seurat packages [41,45]. Peaks were annotated as genomic ranges, and a chromatin assay was created using the GRCm39 genome reference. After normalization using TF-IDF and dimensionality reduction via SVD (LSI), UMAP was performed using components 2 to 30. Clusters were identified using the Louvain algorithm. Gene annotations were retrieved from Ensembl (release 110, mm39). Quality metrics, including transcription start site (TSS) enrichment, nucleosome signal, and blacklist region content, were computed. Cells were filtered based on standard thresholds: TSS enrichment > 2, nucleosome signal < 2, and 100,000 > fragments > 800. Motif accessibility for the AR motif (JASPAR ID: MA0007.3) was quantified using the chromVAR R package (v1.26.0) [24].

### 4.10. Statistical Analysis

Statistical comparisons between experimental groups were conducted using Wilcoxon rank-sum tests for differential gene expression (Seurat). Significance thresholds were set at adjusted *p* < 0.05. As scRNA-seq data are inherently non-normally distributed, non-parametric statistical tests (e.g., Wilcoxon rank-sum test) were applied without assuming normality. Module scores and enrichment *p*-values were calculated using appropriate non-parametric tests. For comparisons of cell-type composition between groups, binomial tests were applied to assess the significance of proportion differences. Sample sizes were based on prior experience with this model; a minimum of 15 mice per group was necessary to obtain sufficient viable cells for scRNA-seq and robust representation of dermal populations [20].

## Data Availability

The scRNA-seq data generated and analyzed in this study have been deposited in the NCBI Gene Expression Omnibus (GEO) under accession number GSE295410 (https://www.ncbi.nlm.nih.gov/geo/query/acc.cgi?acc=GSE295410 (accessed on 6 July 2025)). These data are publicly available and can be accessed without restriction. All datasets necessary to interpret, verify, and extend the findings of this article are included in the GEO submission.

## Supplementary Materials

The following supporting information can be downloaded at: https://www.mdpi.com/article/10.3390/ijms26146568/s1.

## References

[1] Adil A., Godwin M. (2017). The effectiveness of treatments for androgenetic alopecia: A systematic review and meta-analysis. J. Am. Acad. Dermatol..

[2] Manabe M., Tsuboi R., Itami S., Osada S.I., Amoh Y., Ito T., Inui S., Ueki R., Ohyama M., Kurata S. (2018). Guidelines for the diagnosis and treatment of male-pattern and female-pattern hair loss, 2017 version. J. Dermatol..

[3] Aukerman E.L., Jafferany M. (2023). The psychological consequences of androgenetic alopecia: A systematic review. J. Cosmet. Dermatol..

[4] Price V.H., Roberts J.L., Hordinsky M., Olsen E.A., Savin R., Bergfeld W., Fiedler V., Lucky A., Whiting D.A., Pappas F. (2000). Lack of efficacy of finasteride in postmenopausal women with androgenetic alopecia. J. Am. Acad. Dermatol..

[5] Hochfeld L.M., Keller A., Anhalt T., Fricker N., Nöthen M.M., Heilmann-Heimbach S. (2019). Insights into Male Androgenetic Alopecia: Differential Gene Expression Profiling of Plucked Hair Follicles and Integration with Genetic Data. J. Investig. Dermatol..

[6] Zhang Y., Huang J., Fu D., Liu Z., Wang H., Wang J., Qu Q., Li K., Fan Z., Hu Z. (2021). Transcriptome Analysis Reveals an Inhibitory Effect of Dihydrotestosterone-Treated 2D- and 3D-Cultured Dermal Papilla Cells on Hair Follicle Growth. Front. Cell Dev. Biol..

[7] Zheng M., Kim M.H., Park S.G., Kim W.S., Oh S.H., Sung J.H. (2024). CXCL12 Neutralizing Antibody Promotes Hair Growth in Androgenic Alopecia and Alopecia Areata. Int. J. Mol. Sci..

[8] Yu N., Hu T., Yang H., Zhang L., Zhu L., Zhou X., Xiang F., Yang X., Li Y. (2023). Androgen receptor inhibits the hair follicle induction potential of dermal papilla cells by binding with Tcf4 at the A574 binding site. Genes Dis..

[9] Lolli F., Pallotti F., Rossi A., Fortuna M.C., Caro G., Lenzi A., Sansone A., Lombardo F. (2017). Androgenetic alopecia: A review. Endocrine.

[10] Lai J.J., Lai K.P., Chuang K.H., Chang P., Yu I.C., Lin W.J., Chang C. (2009). Monocyte/macrophage androgen receptor suppresses cutaneous wound healing in mice by enhancing local TNF-alpha expression. J. Clin. Investig..

[11] Miao Y., Qu Q., Jiang W., Liu X.M., Shi P.L., Fan Z.X., Du L.J., Wang G.F., Liu X.N., Guo Z.H. (2018). Identification of Functional Patterns of Androgenetic Alopecia Using Transcriptome Profiling in Distinct Locations of Hair Follicles. J. Investig. Dermatol..

[12] Griggs J., Trüeb R.M., Dias M.F.R.G., Hordinsky M., Tosti A. (2021). Fibrosing alopecia in a pattern distribution. J. Am. Acad. Dermatol..

[13] Tao N., Sun Q., Ying Y., Wang Y., Gao J. (2025). Dermal T cell immunity and key regulatory signaling pathways: Implications in immune-mediated alopecia and hair regeneration. Genes Dis..

[14] Zheng M., An S., Park I.G., Kim J., Kim W.S., Noh M., Sung J.H. (2024). Differential Expression of CXCL12 in Human and Mouse Hair: Androgens Induce CXCL12 in Human Dermal Papilla and Dermal Sheath Cup. Int. J. Mol. Sci..

[15] Werner L., Guzner-Gur H., Dotan I. (2013). Involvement of CXCR4/CXCR7/CXCL12 Interactions in Inflammatory bowel disease. Theranostics.

[16] Daniel S.K., Seo Y.D., Pillarisetty V.G. (2020). The CXCL12-CXCR4/CXCR7 axis as a mechanism of immune resistance in gastrointestinal malignancies. Semin. Cancer Biol..

[17] Cuesta-Margolles G., Schlecht-Louf G., Bachelerie F. (2025). ACKR3 in Skin Homeostasis, an Overlooked Player in the CXCR4/CXCL12 Axis. J. Investig. Dermatol..

[18] García-Cuesta E.M., Santiago C.A., Vallejo-Díaz J., Juarranz Y., Rodríguez-Frade J.M., Mellado M. (2019). The Role of the CXCL12/CXCR4/ACKR3 Axis in Autoimmune Diseases. Front. Endocrinol..

[19] Janssens R., Struyf S., Proost P. (2018). Pathological roles of the homeostatic chemokine CXCL12. Cytokine Growth Factor Rev..

[20] An S., Zheng M., Park I.G., Park S.G., Noh M., Sung J.H. (2024). Humanized CXCL12 antibody delays onset and modulates immune response in alopecia areata mice: Insights from single-cell RNA sequencing. Front. Immunol..

[21] Szklarczyk D., Kirsch R., Koutrouli M., Nastou K., Mehryary F., Hachilif R., Gable A.L., Fang T., Doncheva N.T., Pyysalo S. (2023). The STRING database in 2023: Protein-protein association networks and functional enrichment analyses for any sequenced genome of interest. Nucleic Acids Res..

[22] Jin S., Guerrero-Juarez C.F., Zhang L., Chang I., Ramos R., Kuan C.H., Myung P., Plikus M.V., Nie Q. (2021). Inference and analysis of cell-cell communication using CellChat. Nat. Commun..

[23] Zhang C., Wang D., Dowell R., Yi R. (2023). Single cell analysis of transcriptome and open chromatin reveals the dynamics of hair follicle stem cell aging. Front. Aging.

[24] Schep A.N., Wu B., Buenrostro J.D., Greenleaf W.J. (2017). chromVAR: Inferring transcription-factor-associated accessibility from single-cell epigenomic data. Nat. Methods.

[25] Browaeys R., Saelens W., Saeys Y. (2020). NicheNet: Modeling intercellular communication by linking ligands to target genes. Nat. Methods.

[26] Greco V., Chen T., Rendl M., Schober M., Pasolli H.A., Stokes N., Cruz-Racelis J.D., Fuchs E. (2009). A two-step mechanism for stem cell activation during hair regeneration. Cell Stem Cell.

[27] Hagner A., Shin W., Sinha S., Alpaugh W., Workentine M., Abbasi S., Rahmani W., Agabalyan N., Sharma N., Sparks H. (2020). Transcriptional Profiling of the Adult Hair Follicle Mesenchyme Reveals R-spondin as a Novel Regulator of Dermal Progenitor Function. iScience.

[28] Plikus M.V., Mayer J.A., de la Cruz D., Baker R.E., Maini P.K., Maxson R., Chuong C.M. (2008). Cyclic dermal BMP signalling regulates stem cell activation during hair regeneration. Nature.

[29] Wu P., Zhang Y., Xing Y., Xu W., Guo H., Deng F., Ma X., Li Y. (2019). The balance of Bmp6 and Wnt10b regulates the telogen-anagen transition of hair follicles. Cell Commun. Signal..

[30] Miyake K., Ito J., Takahashi K., Nakabayashi J., Brombacher F., Shichino S., Yoshikawa S., Miyake S., Karasuyama H. (2024). Single-cell transcriptomics identifies the differentiation trajectory from inflammatory monocytes to pro-resolving macrophages in a mouse skin allergy model. Nat. Commun..

[31] Kwack M.H., Hamida O.B., Kim M.K., Kim M.K., Sung Y.K. (2023). Establishment and characterization of matched immortalized human frontal and occipital scalp dermal papilla cell lines from androgenetic alopecia. Sci. Rep..

[32] Jung Y.H., Chae C.W., Choi G.E., Shin H.C., Lim J.R., Chang H.S., Park J., Cho J.H., Park M.R., Lee H.J. (2022). Cyanidin 3-O-arabinoside suppresses DHT-induced dermal papilla cell senescence by modulating p38-dependent ER-mitochondria contacts. J. Biomed. Sci..

[33] Herskind C., Sticht C., Sami A., Giordano F.A., Wenz F. (2021). Gene Expression Profiles Reveal Extracellular Matrix and Inflammatory Signaling in Radiation-Induced Premature Differentiation of Human Fibroblast in vitro. Front. Cell Dev. Biol..

[34] Ma Z., Zhou F., Jin H., Wu X. (2024). Crosstalk between CXCL12/CXCR4/ACKR3 and the STAT3 Pathway. Cells.

[35] Liang Y., Hu Y., Zhang J., Song H., Zhang X., Chen Y., Peng Y., Sun L., Sun Y., Xue R. (2024). Dynamic pathological analysis reveals a protective role against skin fibrosis for TREM2-dependent macrophages. Theranostics.

[36] Wang E.C.E., Dai Z., Ferrante A.W., Drake C.G., Christiano A.M. (2019). A Subset of TREM2+ Dermal Macrophages Secretes Oncostatin M to Maintain Hair Follicle Stem Cell Quiescence and Inhibit Hair Growth. Cell Stem Cell.

[37] Ikawa T., Miyagawa T., Fukui Y., Toyama S., Omatsu J., Awaji K., Norimatsu Y., Watanabe Y., Yoshizaki A., Sato S. (2021). Association of serum CXCL12 levels with arthropathy in patients with systemic sclerosis. Int. J. Rheum. Dis..

[38] Meller S., Gilliet M., Homey B. (2009). Chemokines in the pathogenesis of lichenoid tissue reactions. J. Investig. Dermatol..

[39] Nagler A., Labopin M., Shimoni A., Niederwieser D., Mufti G.J., Zander A.R., Arnold R., Greinix H., Cornelissen J.J., Jackson G.H. (2012). Mobilized peripheral blood stem cells compared with bone marrow as the stem cell source for unrelated donor allogeneic transplantation with reduced-intensity conditioning in patients with acute myeloid leukemia in complete remission: An analysis from the Acute Leukemia Working Party of the European Group for Blood and Marrow Transplantation. Biol. Blood Marrow Transplant..

[40] Arganda-Carreras I., Kaynig V., Rueden C., Eliceiri K.W., Schindelin J., Cardona A., Seung H.S. (2017). Trainable Weka Segmentation: A machine learning tool for microscopy pixel classification. Bioinformatics.

[41] Stuart T., Butler A., Hoffman P., Hafemeister C., Papalexi E., Mauck W.M., Hao Y., Stoeckius M., Smibert P., Satija R. (2019). Comprehensive Integration of Single-Cell Data. Cell.

[42] Lee K.J., An S., Kim M.Y., Kim S.M., Jeong W.I., Ko H.J., Yang Y.M., Noh M., Han Y.H. (2023). Hepatic TREM2+ macrophages express matrix metalloproteinases to control fibrotic scar formation. Immunol. Cell Biol..

[43] McGinnis C.S., Murrow L.M., Gartner Z.J. (2019). DoubletFinder: Doublet Detection in Single-Cell RNA Sequencing Data Using Artificial Nearest Neighbors. Cell Syst..

[44] Joost S., Annusver K., Jacob T., Sun X., Dalessandri T., Sivan U., Sequeira I., Sandberg R., Kasper M. (2020). The Molecular Anatomy of Mouse Skin during Hair Growth and Rest. Cell Stem Cell.

[45] Stuart T., Srivastava A., Madad S., Lareau C.A., Satija R. (2021). Single-cell chromatin state analysis with Signac. Nat. Methods.

