# Supplementary material for: CXCL12 Drives Reversible Fibroimmune Remodeling in Androgenetic Alopecia Revealed by Single-Cell RNA Sequencing
Source: Int J Mol Sci. 2025 Jul 8;26(14):6568. doi: 10.3390/ijms26146568 (PMC12294365; doi:10.3390/ijms26146568)
Supplement: Supplementary file 1 [file ijms-26-06568-s001.zip › ijms-3734278-supplementary.pdf]

***Supplemental Information* for**

CXCL12 drives reversible fibroimmune remodeling in androgenetic alopecia revealed by single-cell RNA sequencing

Seungchan An[a,#], Mei Zheng[b,#], In Guk Park[a], Leegu Song[b], Jino Kim[c], Minsoo Noh[a,*], Jong-Hyuk Sung[b,*]

[#] *Equal contribution*

[a] *College of Pharmacy and Natural Products Research Institute, Seoul National University, Seoul, Republic of Korea.*

[b] *Epi Biotech Co., Ltd., Incheon, Republic of Korea.*

[c] *New Hair Plastic Surgery Clinic, Seoul, Republic of Korea*

*Co-correspondence: minsoonoh@snu.ac.kr (M Noh), brian99@epibiotech.com (JH Sung)

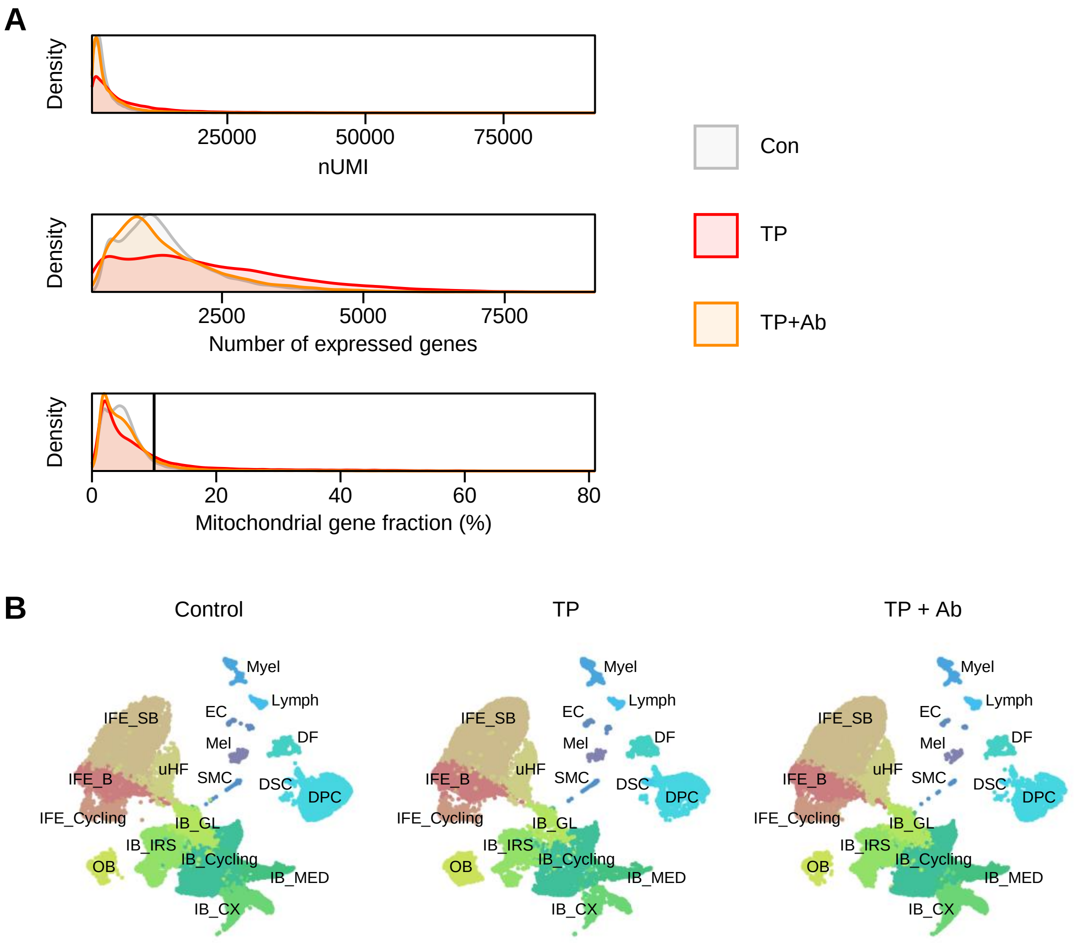

Figure S1. Quality control of scRNA-seq data. (A) Initial quality control filtering. Cells expressing between 200 and 8,000 genes, and with mitochondrial gene expression fraction below 10%, passed the initial quality control filter. (B) UMAP representation of scRNA-seq results from Con, TP, and TP+Ab groups, colored by cell type annotations.

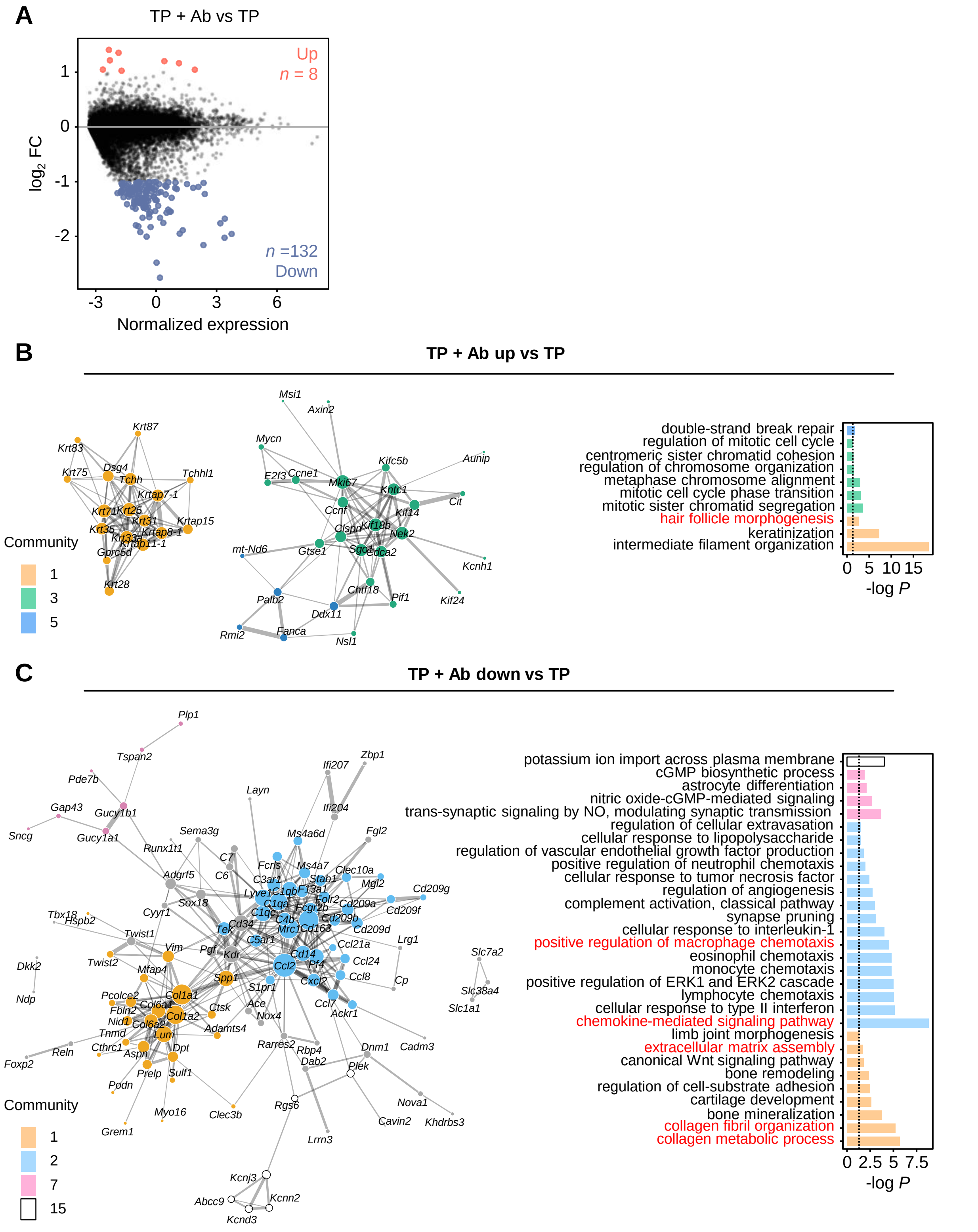

Figure S2. Functional enrichment of DEGs altered by CXCL12 Ab treatment. (A) MA plot of TP+Ab vs TP with DEGs defined as |$\log_2$ fold change (FC)| ≥ 1. (B) GO analysis of genes upregulated in TP+Ab vs TP. DEGs were identified based on $\log_2$ fold change and adjusted $P < 0.05$. The top 100 upregulated genes (by $\log_2$ FC) were used to construct a STRING protein-protein interaction network (left). In the network, vertex (node) size reflects the number of connections (node degree), and edge thickness corresponds to the STRING combined interaction score. Gene communities were defined using the Girvan-Newman algorithm. GO biological process enrichment analysis (adjusted $P < 0.05$) was performed separately for each community (right). (C) Same as (B), but for genes downregulated in TP+Ab vs TP, based on the top 100 downregulated DEGs.

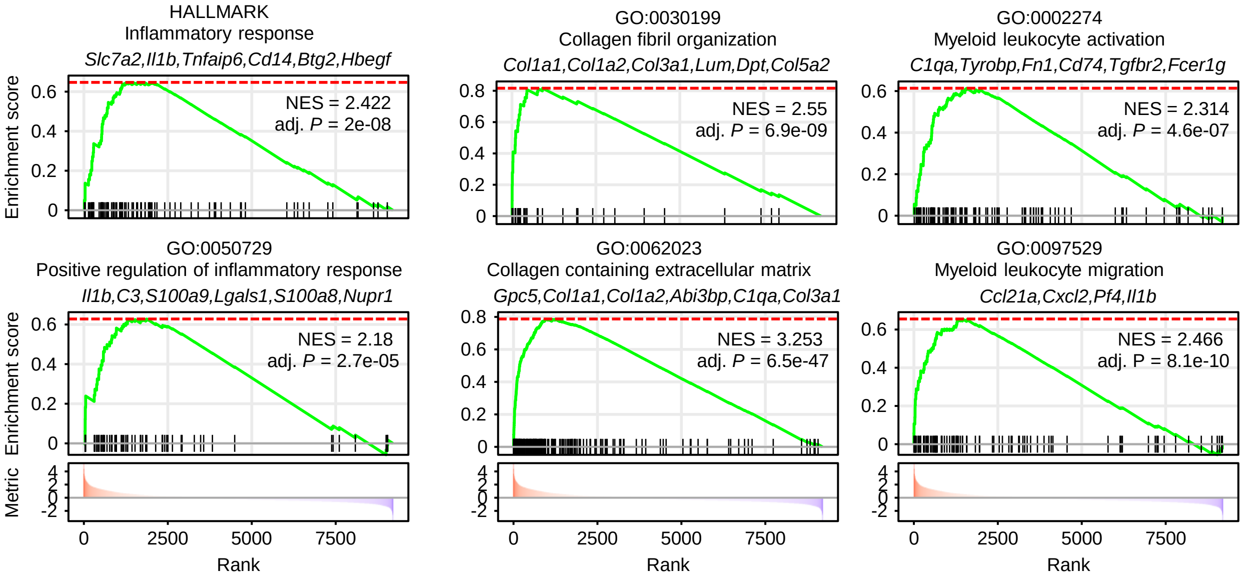

Figure S3. GSEA demonstrating reversal effect of CXCL12 blockade. Input for GSEA was defined as $\log_2$ FC [TP / Con] – $\log_2$ FC [TP+Ab / TP], representing reversal magnitude.

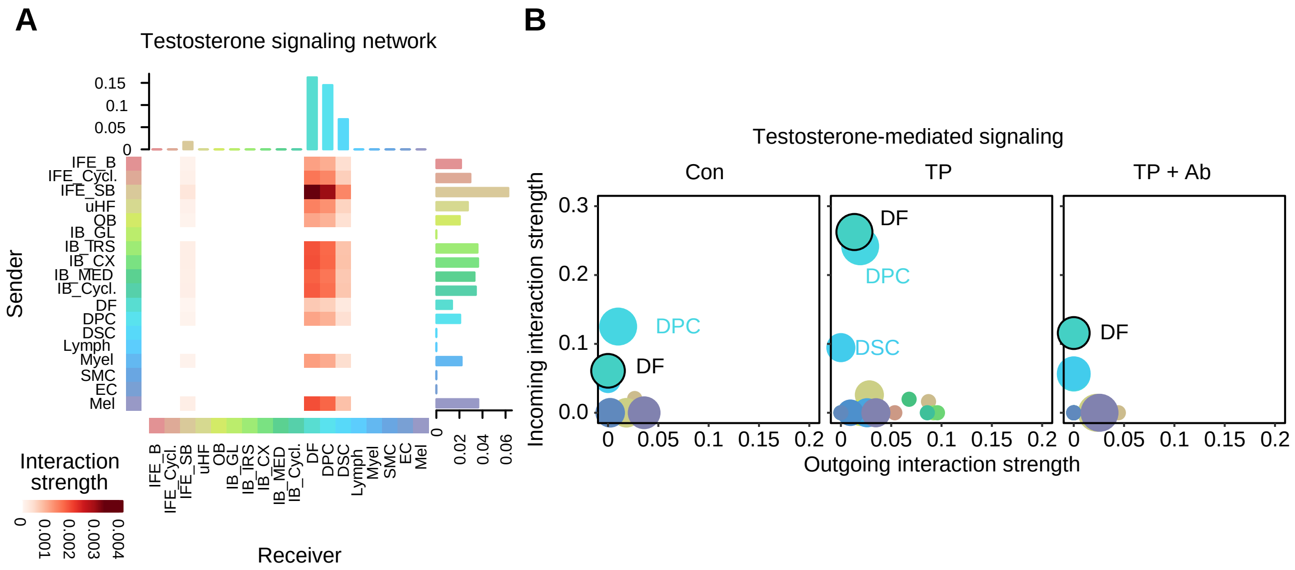

Figure S4. Cell–cell communication analysis of the testosterone signaling network. (A) CellChat analysis of the testosterone signaling pathway across skin cell types. Interaction strength was calculated based on the expression levels of the ligand gene in the sender cell and the corresponding receptor gene in the receiver cell. (B) Testosterone signaling intensity from CellChat analysis. Dot size represents the number of significant interactions.

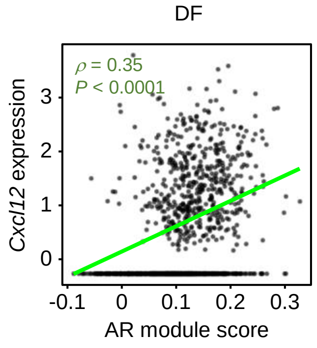

Figure S5. Spearman correlation of AR module score with *Cxcl12* expression in DFs. Correlation was calculated nonparametrically using Spearman's method.

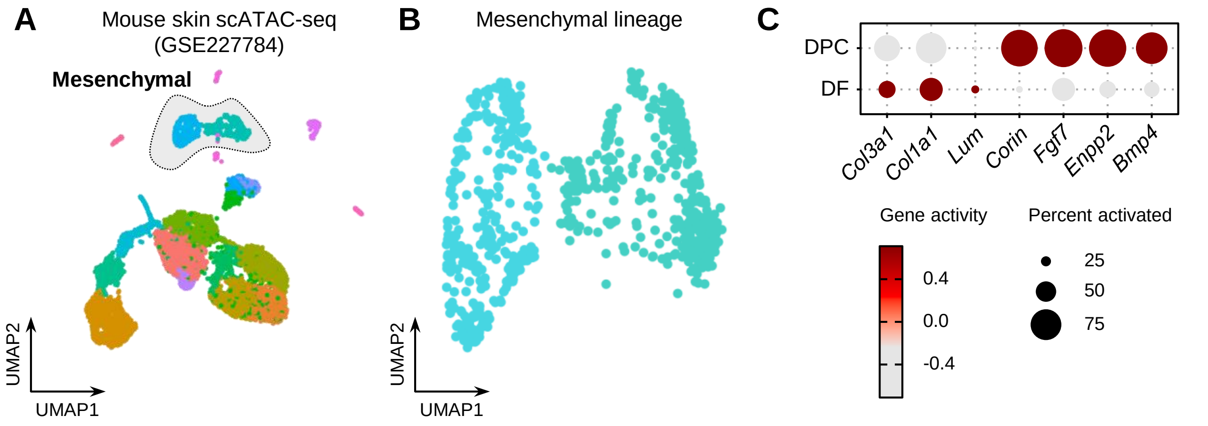

Figure S6. scATAC-seq analysis of mesenchymal subtypes in mouse skin. (A) UMAP representation of scATAC-seq data from GSE227784, colored by Louvain clusters. The peak-by-barcode matrix was normalized using term frequency-inverse document frequency (TF-IDF), followed by singular value decomposition (SVD) for dimensionality reduction. UMAP was generated using components 2 through 30 of the latent semantic indexing (LSI) space. (B) Zoomed-in UMAP view of mesenchymal cells, highlighting subclusters annotated as DF and DPC based on gene activity scores. (C) Dot plot displaying gene activity levels of canonical marker genes used for identifying DF and DPC subtypes.

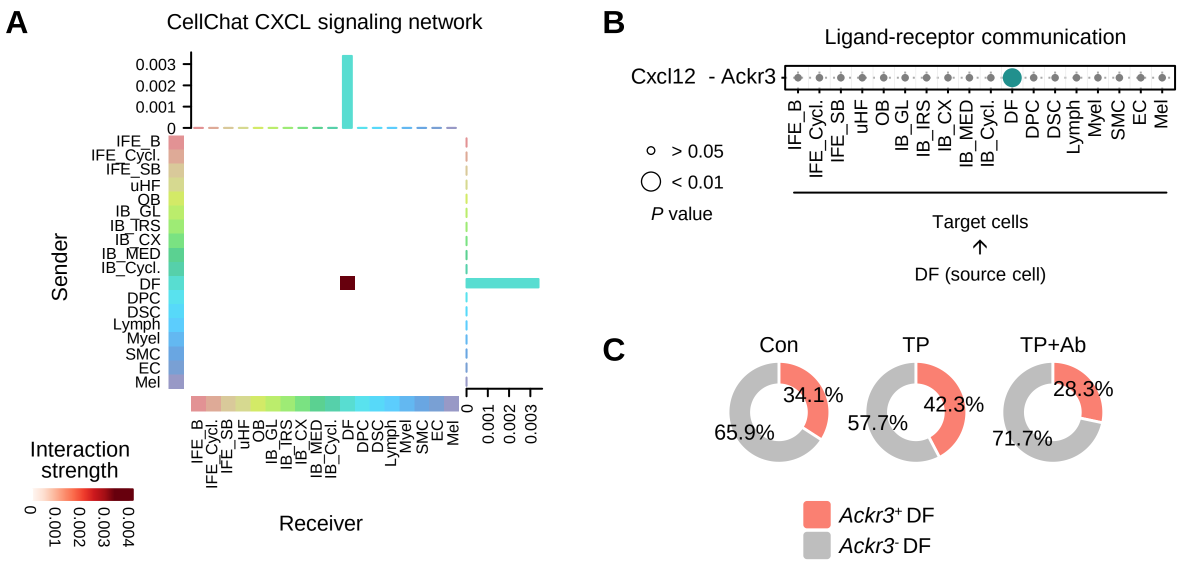

Figure S7. Cell–cell communication analysis of the CXCL12 signaling network. (A) CellChat analysis of the CXCL signaling pathway across skin cell types. Interaction strength was calculated based on the expression levels of the ligand gene in the sender cell and the corresponding receptor gene in the receiver cell. (B) Dot plot showing statistically significant ligand-receptor pairs mediating CXCL12 signaling from DFs to target cells. Dot size represents the *P* value derived from a one-sided permutation test. (C) Proportion of *Ackr3*-expressing cells within the DF subset.

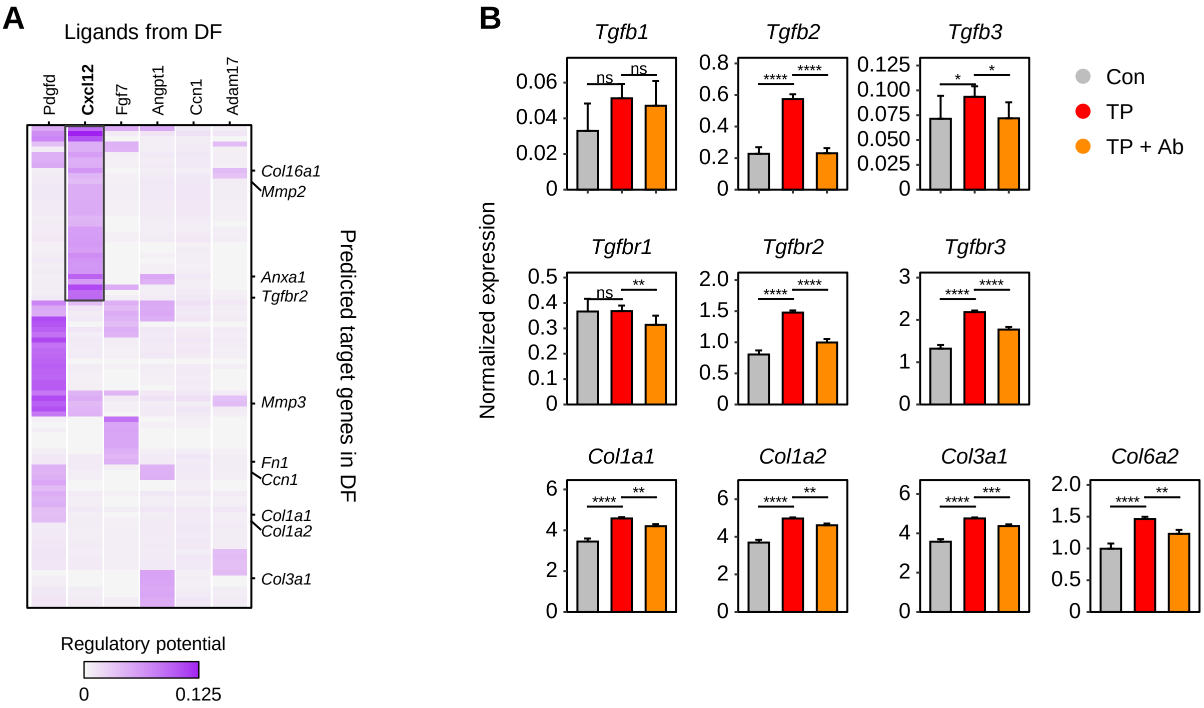

Figure S8. Target genes of DF-derived CXCL12-ACKR3 signaling. (A) NicheNet analysis identified CXCL12 among top DF ligands associated with high regulatory potential for fibrosis-related target genes. Analysis was performed based on differential expression between TP and control. (B) Expression levels of TGF signaling and collagen-related genes across groups. Values are presented as mean ± standard error; Wilcoxon test used for significance (**P* < 0.05, ***P* < 0.01, ****P* < 0.001, and *****P* < 0.0001).

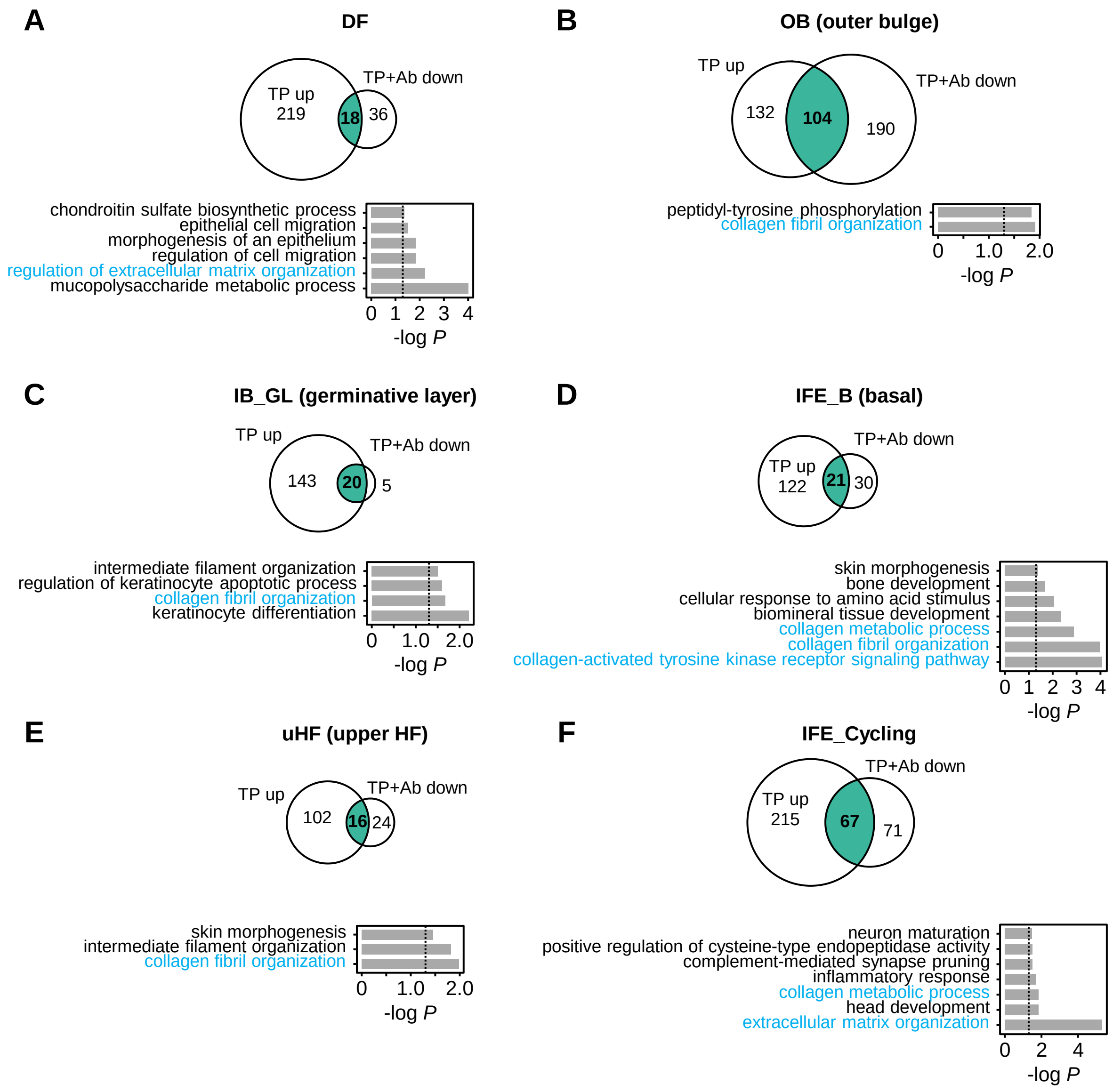

Figure S9. Enrichment analysis of Ab-reversed DEGs across skin cell types. (A) GO biological process enrichment analysis of Ab-reversed DEGs in DF subset (adjusted $P < 0.05$). Ab-reversed DEGs were defined as the intersection of genes upregulated in TP vs Con and downregulated in TP+Ab vs TP. (B-F) Same analysis as in (A), performed for the following subsets: (B) outer bulge (OB), (C) germinative layer of inner bulge (IB_GL), (D) basal interfollicular epidermis (IFE_B), (E) upper hair follicle (uHF), and (F) cycling IFE.

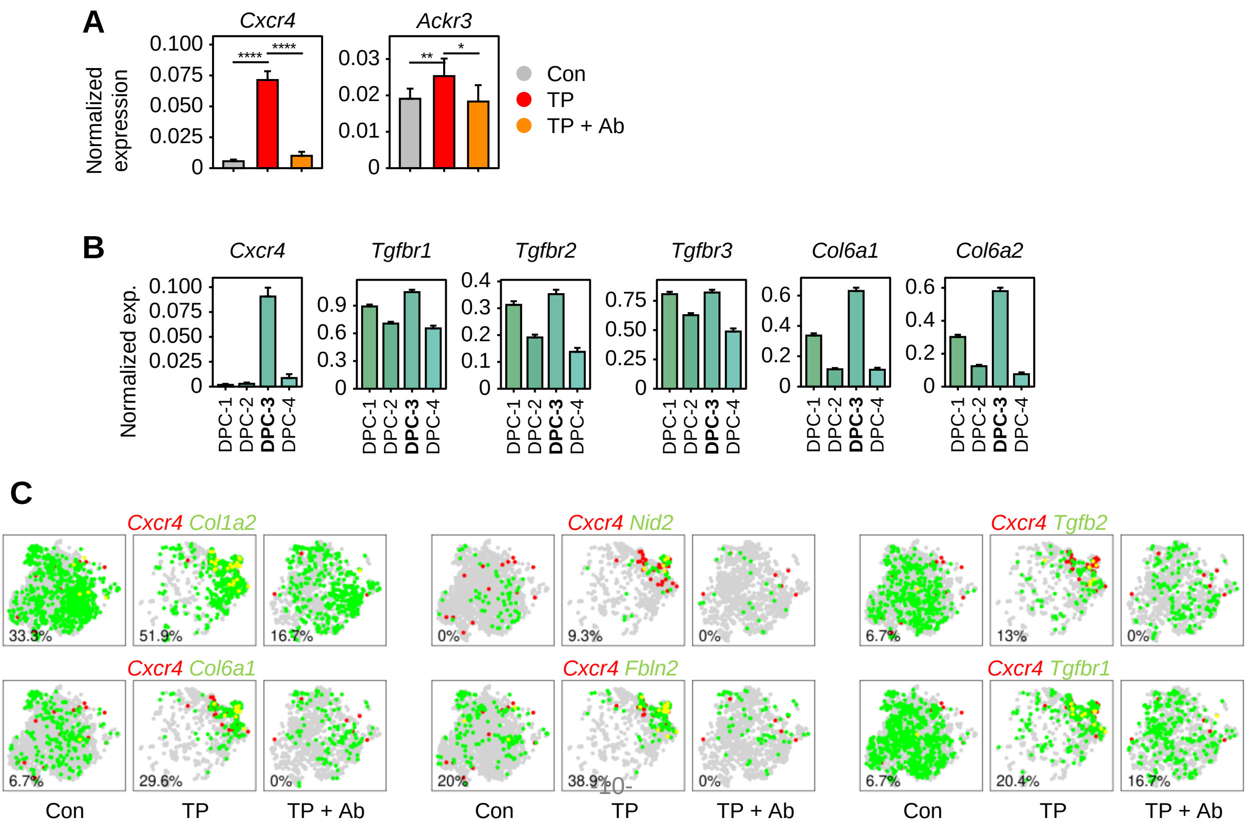

Figure S10. Expression of *Cxcr4* and related genes in DPCs. (A) Mean (± standard error) expression of *Cxcr4* and *Ackr3* in DPCs across groups. Wilcoxon test used for significance (**P* < 0.05, ***P* < 0.01, ****P* < 0.001, and *****P* < 0.0001). (B) Expression levels of *Cxcr4*, *Tgfbr1/2/3*, and *Col6a1/2* in DPC subclusters. (C) Co-expression analysis of *Cxcr4* with ECM and TGF-β genes in DPCs; red = *Cxcr4*$^{+}$, green = specified gene-positive, and yellow = double-positive.

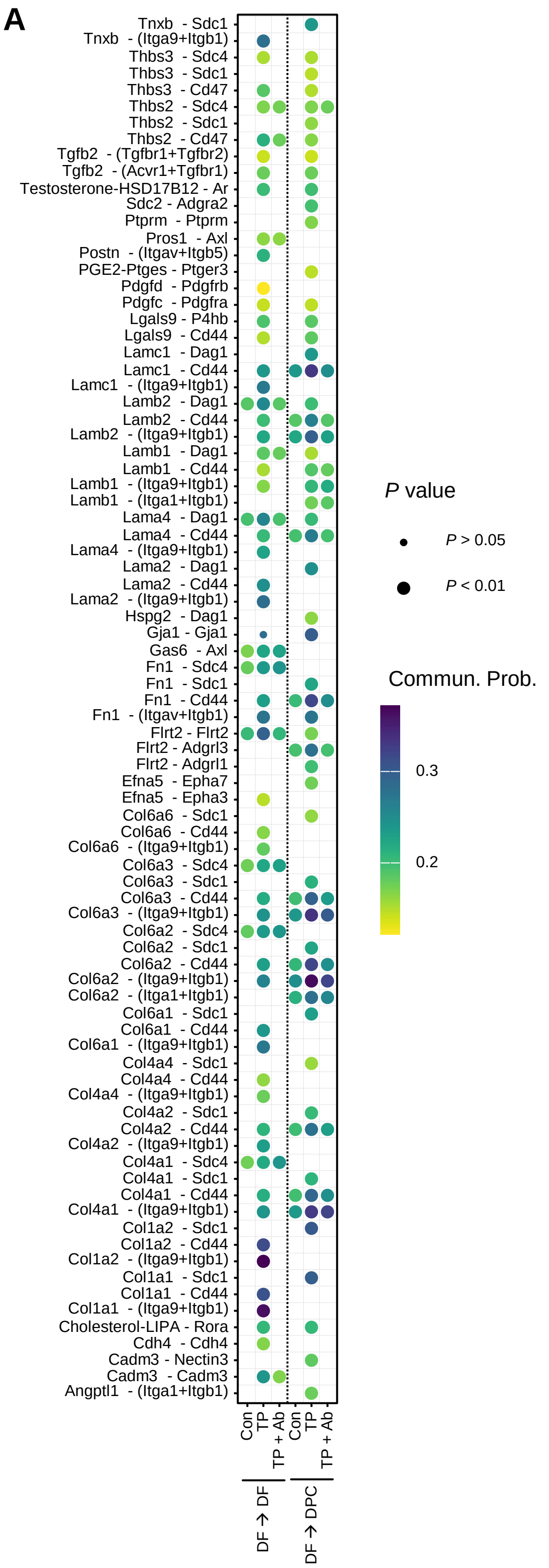

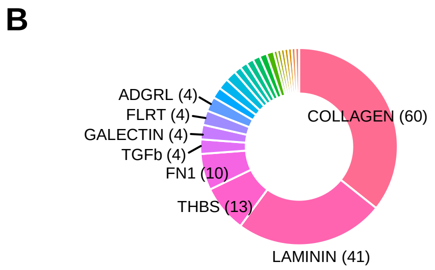

C

**Signaling pathway**	**Adjusted *P* value**
COLLAGEN	< 1e-16
LAMININ	2.39e-09
FN1	9.35e-09
HSPG	3.76e-08
PTPRM	3.76e-08
THBS	5.04e-08

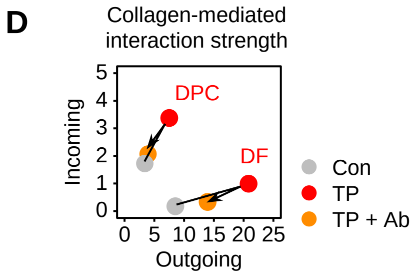

Figure S11. Cell-cell communication between DF and DPC. (A) Dot plot of significant ligand-receptor pairs mediating communication between DFs and DPCs, with emphasis on interactions reversed by CXCL12 blockade. Reversal was defined as a >3-fold change in communication probability. Dot color and size represent the communication probability and *P* value from a one-sided permutation test, respectively. (B) Distribution of ligand-receptor pairs by signaling pathway categories. (C) Enrichment of major signaling pathways in DF-DPC interactions compared to the global CellChat signaling database, assessed by chi-squared test. (D) CellChat analysis of collagen signaling interaction strength between DFs and DPCs.

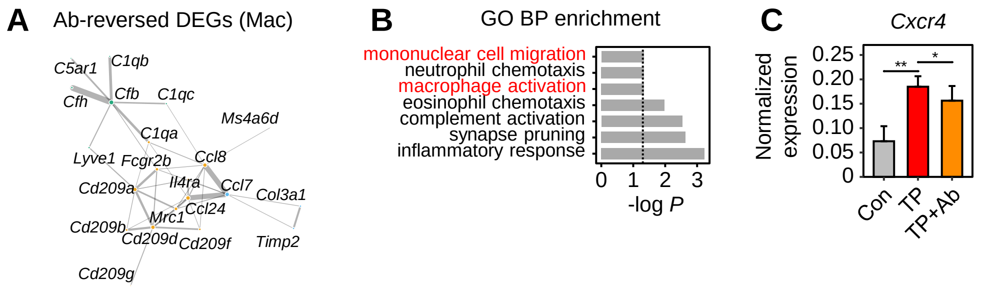

Figure S12. GO enrichment in macrophages. (A-B) STRING network and GO BP enrichment of Ab-reversed DEGs in macrophages. (C) Mean (± standard error) expression level of *Cxcr4* in macrophages across groups. Wilcoxon test; $^{*}P < 0.05$ and $^{**}P < 0.01$.

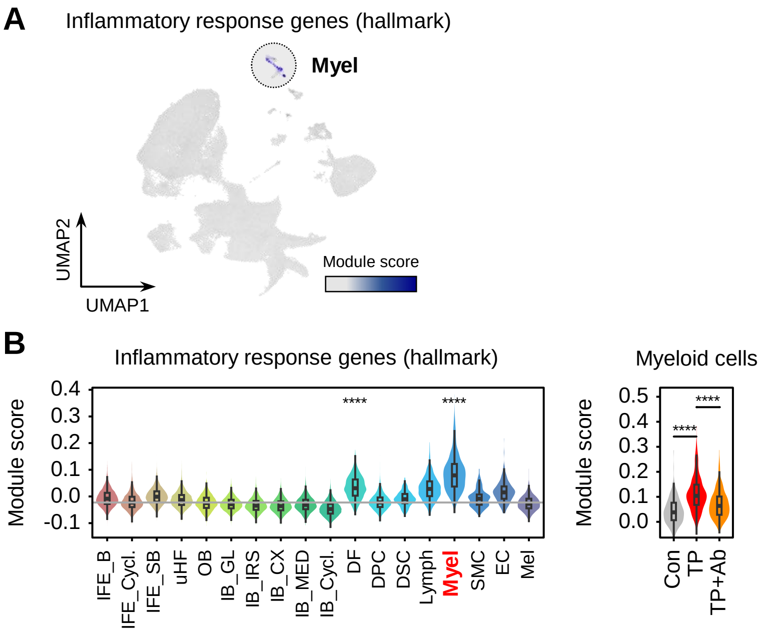

Figure S13. Inflammatory response module scores. (A) UMAP plot colored by inflammatory response module score based on MSigDB Hallmark genes. (B) Violin plots of inflammatory response module score by cell type and group. Wilcoxon test; $^{****}P < 0.0001$.

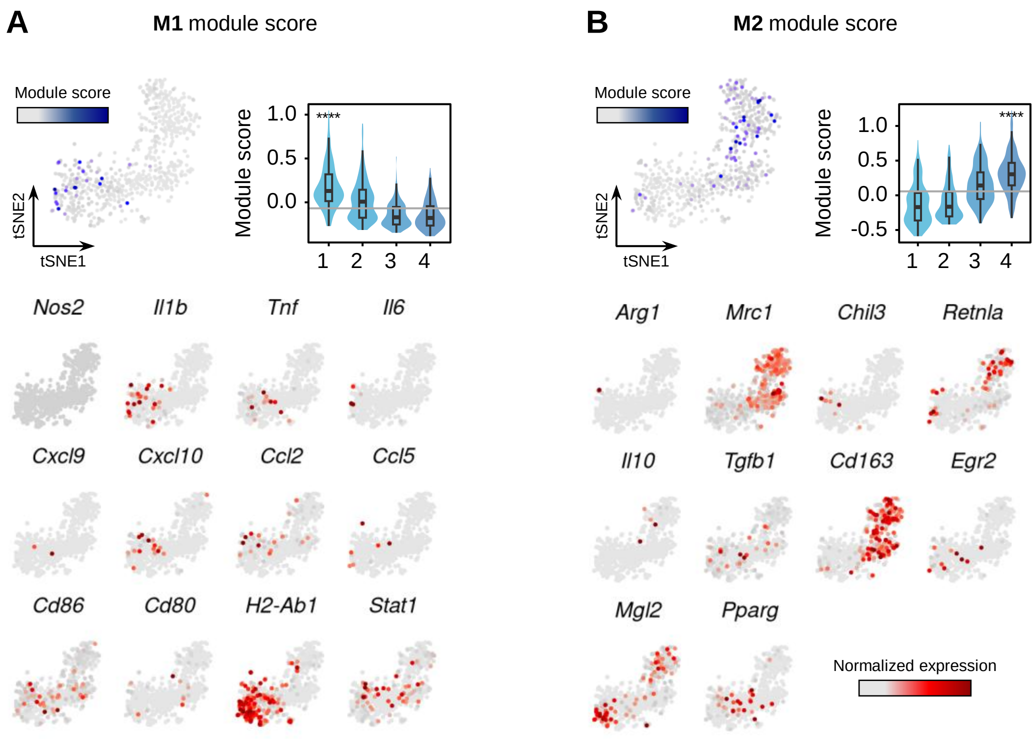

Figure S14. M1 and M2 module scores in macrophage subpopulations. (A) Top: tSNE representation of macrophage subsets colored by M1 module score, alongside violin plots comparing M1 scores across subpopulations. Module scores were calculated using a curated set of M1-associated genes. Wilcoxon test; ****P < 0.0001. Bottom: tSNE plots colored by expression levels of representative M1 marker genes. (B) Same as (A), but for the M2 module score, calculated using selected M2-associated genes.
